# Bioinformatics identification and experimental validation of m6A-related diagnostic biomarkers in the subtype classification of blood monocytes from postmenopausal osteoporosis patients

**DOI:** 10.3389/fendo.2023.990078

**Published:** 2023-03-08

**Authors:** Peng Zhang, Honglin Chen, Bin Xie, Wenhua Zhao, Qi Shang, Jiahui He, Gengyang Shen, Xiang Yu, Zhida Zhang, Guangye Zhu, Guifeng Chen, Fuyong Yu, De Liang, Jingjing Tang, Jianchao Cui, Zhixiang Liu, Hui Ren, Xiaobing Jiang

**Affiliations:** ^1^ Guangzhou University of Chinese Medicine, Guangzhou, China; ^2^ Lingnan Medical Research Center of Guangzhou University of Chinese Medicine, Guangzhou, China; ^3^ The First Affiliated Hospital of Guangzhou University of Chinese Medicine, Guangzhou, China; ^4^ Affiliated Huadu Hospital, Southern Medical University, Guangzhou, China

**Keywords:** postmenopausal osteoporosis, RNA N6-methyladenosine (m6A) modulators, subtype classification, risk prediction, experimental validation

## Abstract

**Background:**

Postmenopausal osteoporosis (PMOP) is a common bone disorder. Existing study has confirmed the role of exosome in regulating RNA N6-methyladenosine (m6A) methylation as therapies in osteoporosis. However, it still stays unclear on the roles of m6A modulators derived from serum exosome in PMOP. A comprehensive evaluation on the roles of m6A modulators in the diagnostic biomarkers and subtype identification of PMOP on the basis of GSE56815 and GSE2208 datasets was carried out to investigate the molecular mechanisms of m6A modulators in PMOP.

**Methods:**

We carried out a series of bioinformatics analyses including difference analysis to identify significant m6A modulators, m6A model construction of random forest, support vector machine and nomogram, m6A subtype consensus clustering, GO and KEGG enrichment analysis of differentially expressed genes (DEGs) between different m6A patterns, principal component analysis, and single sample gene set enrichment analysis (ssGSEA) for evaluation of immune cell infiltration, experimental validation of significant m6A modulators by real-time quantitative polymerase chain reaction (RT-qPCR), etc.

**Results:**

In the current study, we authenticated 7 significant m6A modulators *via* difference analysis between normal and PMOP patients from GSE56815 and GSE2208 datasets. In order to predict the risk of PMOP, we adopted random forest model to identify 7 diagnostic m6A modulators, including FTO, FMR1, YTHDC2, HNRNPC, RBM15, RBM15B and WTAP. Then we selected the 7 diagnostic m6A modulators to construct a nomogram model, which could provide benefit with patients according to our subsequent decision curve analysis. We classified PMOP patients into 2 m6A subtypes (clusterA and clusterB) on the basis of the significant m6A modulators *via* a consensus clustering approach. In addition, principal component analysis was utilized to evaluate the m6A score of each sample for quantification of the m6A subgroups. The m6A scores of patients in clusterB were higher than those of patients in clusterA. Moreover, we observed that the patients in clusterA had close correlation with immature B cell and gamma delta T cell immunity while clusterB was linked to monocyte, neutrophil, CD56dim natural killer cell, and regulatory T cell immunity, which has close connection with osteoclast differentiation. Notably, m6A modulators detected by RT-qPCR showed generally consistent expression levels with the bioinformatics results.

**Conclusion:**

In general, m6A modulators exert integral function in the pathological process of PMOP. Our study of m6A patterns may provide diagnostic biomarkers and immunotherapeutic strategies for future PMOP treatment.

## Introduction

Postmenopausal osteoporosis (PMOP) is a common bone disorder associated with ageing occurring in postmenopausal women, which is resulted from bone mass decrease and structural changes in bone tissue due to estrogen deficiency, resulting in increased bone fragility and susceptibility to fracture, as well as pain, bone deformation, comorbidities and even death caused by fracture (1–3). It is reported that approximately 50% of women experience at least one PMOP-related fracture (4). Existing drugs including vitamin D, calcium, denosumab, teriparatide, and bisphosphonates serve as recommended therapies for the treatment of PMOP (5), but long-term use of them trigger some side effects causing rapid bone loss and increasing the risks of the jaw osteonecrosis, atypical femoral fractures, and multiple rebound-related vertebral fractures (6). Therefore, PMOP still remains clinically not well managed (7). PMOP seriously impacts the health and life quality of the elderly and even shortens their life expectancy, increasing the financial and social burden on the countries and the families (8). Therefore, it is indispensable and critical to early identify patients at high risk of developing PMOP. Mounting evidence on the extensive developments in PMOP research shows that PMOP is a complicated disease of great heterogeneity that involves genetic changes (9). Hence, early identification and effective prevention of high-risk patients from a genetic perspective will exert a profound influence on the epidemiological control of PMOP.

Notably, recent studies have reported the promise of exosomes as potential therapies in osteoporosis (10, 11). Exosomes are small single-membrane organelles between 40 and 160 nm in diameter (12), which can carry a variety of cargos, such as lipids, proteins, glycoconjugates, and nucleic acids (13). Exosomes can transmit signals or molecules between cells and reshape the extracellular matrix by releasing these substances (14). Moreover, exosome can carry circular RNAs (circRNAs) to regulate bone metabolism in PMOP *via* sponging microRNAs (miRNAs), which can control mRNA expression by regulate the interaction with m6A methylation (15). N6-methyladenosine (m6A) is a widespread epigenetic modification that affects the variable splicing, translocation, translation and degradation of mRNA, as well as the epigenetic effects of certain non-coding RNAs (16). As an essential epigenetic modification, m6A modification needs numerous regulatory proteins encoded by writers, erasers, and readers to coorperate together (17). Abnormalities in m6A methylation can lead to a variety of diseases such as obesity, glioblastoma, acute myeloid leukaemia, type 2 diabetes, infertility, neuronal diseases, premature ovarian failure and various malignancies (18, 19). With the further study on m6A, researchers also found that bone marrow mesenchymal stem cells (BMSCs), chondrocytes, osteoblasts, osteoclasts, osteosarcoma, and adipocytes cells are all subject to m6A modification to regulate the methylation of RNA in cells, affecting the transduction of mRNA and/or non-coding RNA associated genes, thus activating cellular signaling pathways and affecting cell cycle and DNA damage repair, which in turn determines the occurrence and development process of musculoskeletal disorders (20–24). Recently, existing researches have verified that m6A modifications exert vital functions on the pathology of PMOP *via* modulating the expression level of m6A-associated genes (25, 26). However, it still stays unclear on the roles of m6A modulators derived from serum exosome in PMOP.

**Figure 1:**
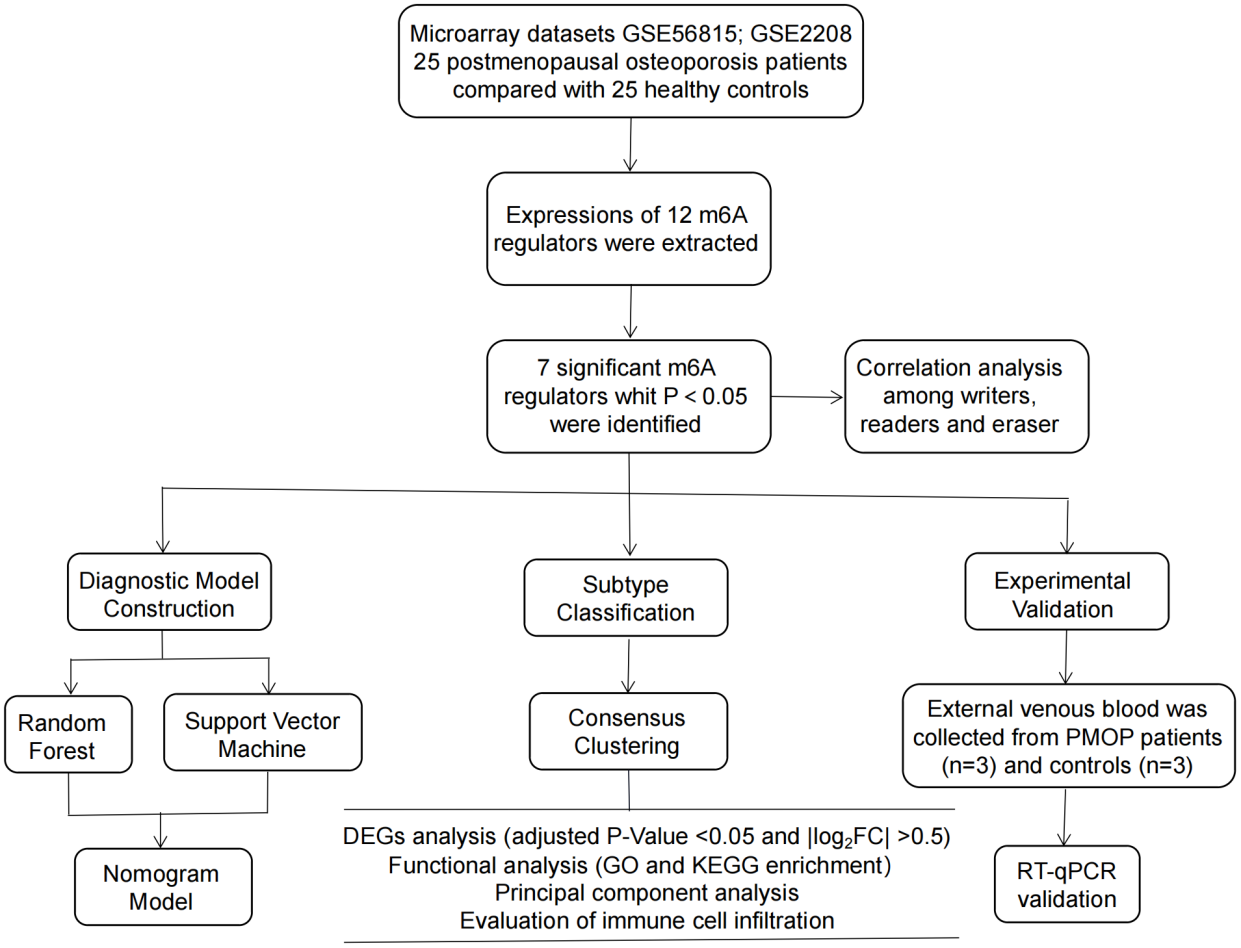
Flow chart of the study design.

In this study, we performed a comprehensive evaluation on the roles of m6A modulators in the diagnostic biomarkers and subtype identification of PMOP on the basis of GSE56815 and GSE2208 datasets with monocyte samples. We developed a PMOP susceptibility prediction gene model based on seven candidate m6A modulators including FMR1, FTO, WTAP, YTHDC2, HNRNPC, RBM15 and RBM15B, and found that the model provided good clinical benefits for patients. Our RT-qPCR experiments further validated these m6A modulators, exhibiting consistent expression levels with the bioinformatics results. Additionally, we excavated two different m6A patterns that were closely correlated with immature B cell, gamma delta T cell, CD56dim natural killer cell, monocyte, neutrophil and regulatory T cell immunity, indicating that m6A patterns may be used to identify PMOP and provide subsequent treatment strategies. **Figure 1** displayed the flowchart of study design and process.

## Materials and methods

### Sample retrieval

We collected monocyte samples separated from whole blood of elderly women by retrieving the GEO database (http://www.ncbi.nlm.nih.gov/geo/). The search terms were “BMD”, “Postmenopausal Osteoporosis”, “Gene expression”, “Microarray”, and the datasets were based on the following criteria: (1) each dataset includes at least 10 samples; (2) each dataset includes at least 5 cases in the groups of control and PMOP respectively; and (3) Both raw data and series matrix file can be obtained from the GEO datasets. Two datasets, GSE56815 (27) and GSE2208 (28) were eventually screened, which fully met our criteria. We chose 5 cases of control group and 5 cases of PMOP group from the dataset of GSE2208 as well as 20 cases of PMOP and 20 controls in GSE56815 dataset for subsequent analysis. **Table 1** showed specific information of the corresponding datasets.

**Table 1:** Information for the selected microarray datasets.

GEO Accession Total samples	Selected samples Platform	Source tissue
19 samples	10 samples	blood monocytes
GSE2208 Sample types:	GPL96 Sample types:	
10 high BMD	5 PreH BMD (Control)	
9 low BMD	5 postL BMD (PMOP)	
80 samples	40 samples	blood monocytes
GSE56815 Sample types:	GPL96 Sample types:	
40 high BMD	20 PreH BMD (Control)	
40 low BMD	20 postL BMD (PMOP)	

BMD, bone mineral density; PreH BMD: Premenopausal High BMD; postL BMD: Postmenopausal Low BMD.

### Data acquisition

We downloaded the annotated R package *via* Bioconductor (http://bioconductor.org/) to convert microarray probes to symbols in R (v4.1.2) software (Statistics Department of the University of Auckland, New Zealand). After data preparation, we carried out consolidation of the two datasets *via* SVA batch difference processing of combat and obtained the final dataset which contained 25 controls and 25 PMOP cases. Differential m6A madulators were identified from the dataset by difference analysis of control and PMOP cases using the R package of Limma. The screening thresholds to determine the significant m6A madulators were *P*-Value <0.05 and |log_2_ fold change (FC)| >0 (29).

### Model construction

We established random forest (RF) and support vector machine (SVM) models as training models to evaluate the PMOP occurrence, which were detected by “Reverse cumulative distribution of residual”, receiver operating characteristic (ROC) curve, and “Boxplots of residual”. In RF model, we used the R package of “RandomForest” to build an RF model to screen candidate m6A modulators with importance score (Mean Decrease Gini)>2. In SVM model, n stands for the number of m6A modulators and every data dot is presented as a dot in an n-dimensional space. We then selected an optimal hyperplane that distinguishes these two groups of control and PMOP very well (30). We then used the R package of “rms” to establish a nomogram model to predict the prevalence of PMOP patients according to screened candidate m6A modulators. We utilized the calibration curve to assess how well our predicted values align with reality. We also carried out decision curve analysis (DCA) to draw a clinical impact curve and assess whether decisions based on the model produced benefit to patients (31).

### Subtype classification

Consensus clustering is a resampling-based algorithm that identifies each member and its subcluster number, and verifies the rationality of the clusters (31). Using the R package of “ConsensusClusterPlus”, a consensus clustering method was conducted to identify different m6A patterns on the basis of significant m6A moderators (32).

### Classification of differentially expressed genes between different m6a patterns and GO and KEGG enrichment analysis

We utilized Limma package to identify differentially expressed genes (DEGs) between different m6A patterns with the threshold of adjusted *P*-Value <0.05 and |log_2_ FC| >0.5. Next, we used the R package of “clusterProfiler” to perform GO and KEGG analyses so as to investigate the possible mechanism of the DEGs involved in PMOP (33).

### Calculation of the m6A score

We utilized principal component analysis to calculate the m6A score for each sample for quantification of the m6A patterns, with the m6A score evaluated based on the following formula: m6A score = PC1_i_, where PC1 denotes principal component 1, and i denotes significant m6A gene expression (34).

### Evaluation of immune cell infiltration

We utilized single sample gene set enrichment analysis (ssGSEA) to evaluate the level of immune cell infiltration in the samples from PMOP groups. First, the gene expression levels in the samples were sequenced using ssGSEA to obtain a ranking of gene expression levels. Next, we searched for the significant m6A madulators in the input dataset and then summed their expression levels. According to these evaluations, we obtained the abundance of immune cells in each sample (35).

### Experimental validation by RNA extraction and real-time quantitative polymerase chain reaction

The clinical experiments involved in this paper were authorized by the Ethics Committee of the 1st Affiliated Hospital of GZUCM (No. K[2019]129). In the current research, all patients who participated in this trial provided informed consent at the beginning. Then, external venous blood was drawn from PMOP patients (n=3) and healthy controls (n=3) respectively. The two groups were age-matched. The manipulation of human peripheral blood monocytes (HPBMs) was performed as described previously (36). First, whole blood from patients was put into a 50-mL centrifuge tube, then diluted with 10-mL PBS and gently mixed. Afterwards, we continuously centrifuged the initial blood specimen at 2000 rpm for 20 minutes. When centrifugation was finished, the blood sample was stratified and the leukocyte layer in the center of the sample containing HPBMs was aspirated by pipette and transferred to a single fresh 15 mL centrifuge tube in liquid with 10-15 mL of PBS. Next the solution was centrifuged at 1500 rpm in 10 min and the supernatant was lifted to precipitate and be the wanted HPBMs. HPBMs were inoculated in 6-well plates, and then 1mL of TRIzol reagent was applied to each well for total RNA extraction from the cells. Subsequently, retrotranscription of 1μg of total RNA was done using a cDNA synthesis kit (Takara Inc.Shiga, Japan). 20μL SYBR Green qPCR SuperMix (Takara Inc.) was used for detection of m6A cDNAs and RT-qPCR machine (Bio-Rad, Hercules, CA, USA). The thermal cycling conditions for the final gene amplification were: 95°C for 30s, 40 cycles of 95°C for 5s, and a final step of 60°C for 30s. Quantitative analysis was performed using the 2^ΔΔCT^ method to calculation of the relative expression of each gene. The gene-related detection primers of m6A modulators were compounded by Shanghai Sangon Biotechnology Co.Ltd (China), as shown in **Table 2**.

**Table 2:** Sequences of m6A gene-specific primers used for RT-qPCR.

m6A genes	Sequence (5’->3’)	
	Forward primer	Reverse primer
FTO	ATTCTATCAGCAGTGGCAGC	GGATGCGAGATACCGGAGTG
FMR1	CCTGAACTCAAGGCTTGGCA	TCTCTTCCTCTGTTGGAGCTTTA
YTHDC2	ACGGGGACCAGAGAGAAATG	TTGTTGAGTCGCCCACTTGT
RBM15	ATGCCTTCCCACCTTGTGAG	CAACCAGTTTTGCACGGACA
WTAP	GCTTCTGCCTGGAGAGGATT	GTGTACTTGCCCTCCAAAGC

### Statistical analysis

The correlations among writer, reader and eraser were evaluated *via* linear regression analyses. The differences between groups were calculated through Kruskal-Wallis tests in bioinformatics analysis, while unpaired t-tests with Welch’s correction were utilized in RT-qPCR data analysis. Two-tailed tests were conducted to estimate all parametric analyses with *P*< 0.05 considered as statistical significance. All results were expressed as mean ± standard deviation.

## Results

### Identification of the 12 m6A modulators in PMOP

**Figure 2:**
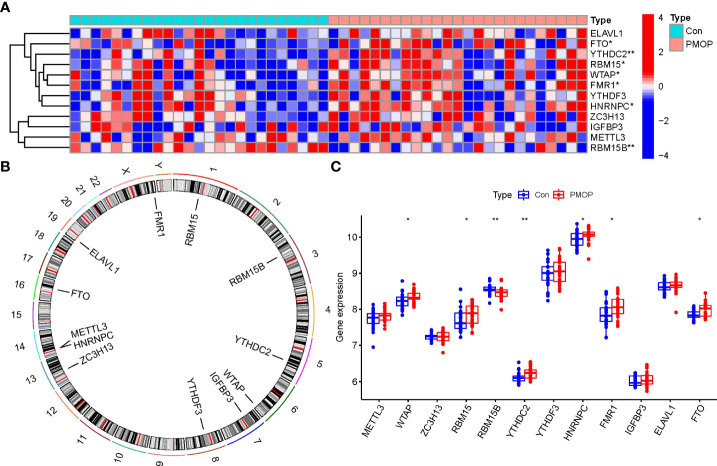
Identification of the 12 m6A modulators in PMOP. **(A)** Expression heat map of the 12 m6A modulators in controls and PMOP cases. **(B)** Chromosomal positions of the 12 m6A modulators. **(C)** Differential expression boxplots of the 12 m6A modulators between controls and PMOP cases. *p < 0.05, and **p < 0.01.

Totally 12 m6A modulators were identified based on difference analysis between controls and PMOP cases. These modulators included one eraser (FTO), five writers (METTL3, ZC3H13, RBM15B, WTAP, and RBM15), and six readers (YTHDC2, ELAVL1, FMR1, YTHDF3, HNRNPC, and IGFBP3). We finally filtrated 7 vital m6A modulators (HNRNPC, YTHDC2, FMR1, FTO, WTAP, RBM15B, and RBM15), which were visualized by a heat map and histogram. We observed that RBM15B expression was decreased in PMOP cases compared to controls, while the other significant m6A regulators displayed the opposite results (**Figures 2A, C**). And we visualized the chromosomal positions of the 12 m6A modulators *via* the “RCircos” package (**Figure 2B**).

### Correlation among writers, readers and eraser in PMOP

**Figure 3:**
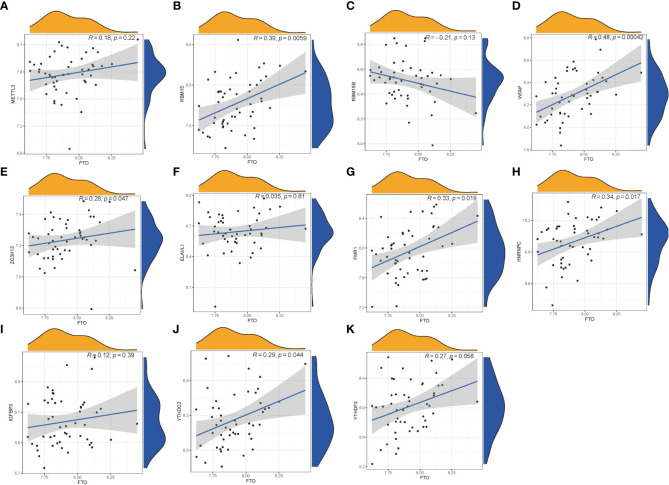
Correlation among Writers, Readers and Eraser in PMOP **(A–K)**. Writer genes: RBM15, RBM15B, METTL3, WTAP, and ZC3H13; reader genes: ELAVL1, FMR1, HNRNPC, IGFBP3, YTHDC2, and YTHDF3; eraser gene: FTO.

We utilized linear regression analyses to investigate whether gene expression levels of writers or readers in PMOP exhibit correlation with the gene expression level of eraser. We observed that the gene expression levels of writers RBM15, WTAP, ZC3H13, and readers FMR1, YTHDC2, and HNRNPC in PMOP cases were positively correlated with eraser gene FTO. The other readers or writers were not significantly linked to eraser gene FTO (**Figure 3**). Thus, we demonstrated different correlations between different writers, readers and eraser.

### Establishment of the RF and SVM models

**Figure 4:**
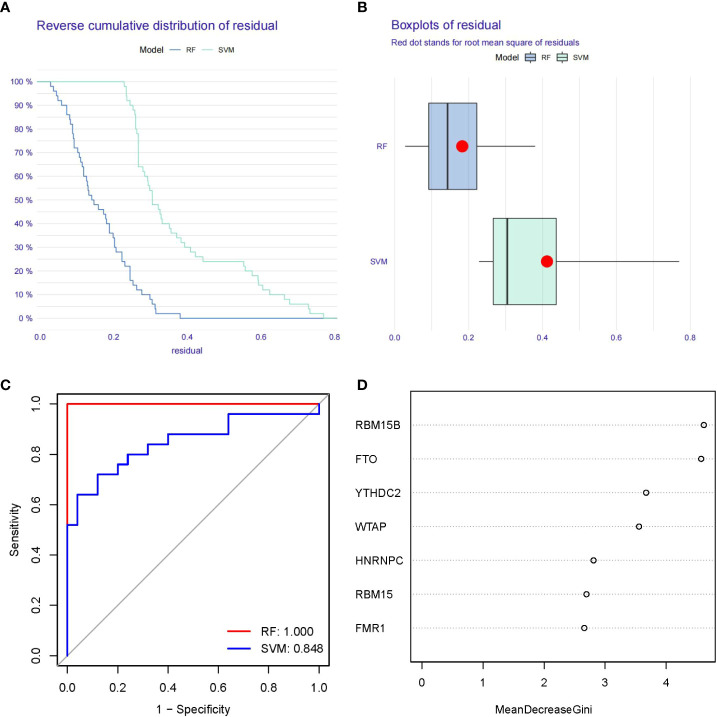
Establishment of the RF and SVM Models. **(A)** Reverse cumulative distribution of residual was constructed to display the residual distribution of RF and SVM models. **(B)** Boxplots of residual was construct to display the residual distribution of RF and SVM models. **(C)** ROC curves indicated the accuracy of the RF and SVM models. **(D)** The importance score of the 7 m6A modulators on the basis of the RF model.

**Figure 4A** showed “Reverse cumulative distribution of residual” and **Figure 4B** presented “Boxplots of residual”, which confirmed that the RF model has the smallest residuals. The residuals for most of the samples in the model are relatively small, suggesting that the RF model is better than the SVM model. Therefore, we determined the RF model to be the most suitable model for the prediction of PMOP occurrence. Then, we plotted ROC curve to estimate the models, and found that the RF model is more accurate than the SVM model according to their AUC values of the ROC curves (**Figure 4C**). Finally, we visualized these 7 significant m6A regulators after ranking them in order of importance and selected m6A regulators with importance score>2 as the candidate genes (**Figure 4D**).

### Establishment of the nomogram model

**Figure 5:**
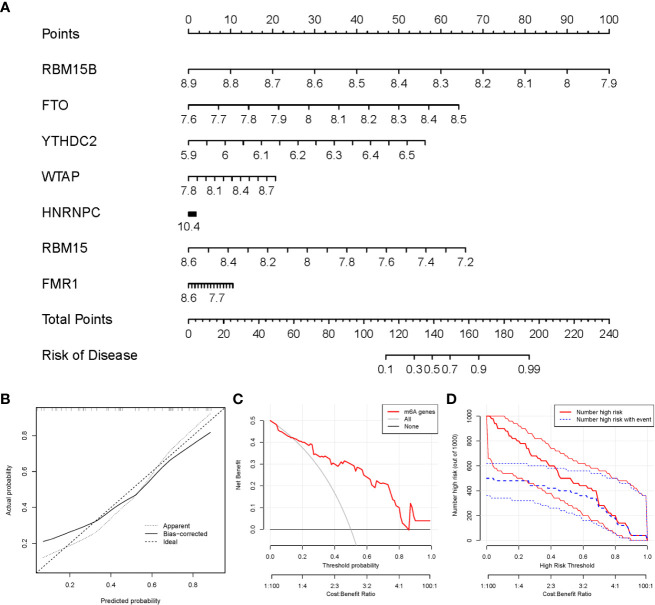
Establishment of the nomogram model. **(A)** The nomogram model was established on the basis of the 7 candidate m6A modulators. **(B)** The calibration curve was utilized to evaluate the predictive accuracy of the nomogram model. **(C)** Decisions on the basis of this nomogram model may be beneficial to PMOP patients. **(D)** The clinical impact curve was used to assess clinical impact of the nomogram model.

We utilized the “rms” package in R to establish a nomogram model of the seven candidate m6A modulators for the prediction of the prevalence of PMOP patients (**Figure 5A**). We observed that the nomogram model exhibits high accuracy of prediction according to calibration curves (**Figure 5B**). The red line in the DCA curve stayed above the gray and black lines from 0 to 1, suggesting that decisions based on the nomogram model may be beneficial to PMOP patients (**Figure 5C**). Moreover, we noticed that the predictive power of the nomogram model was remarkable according to the clinical impact curve (**Figure 5D**).

### Identification of two distinct m6A patterns

**Figure 6:**
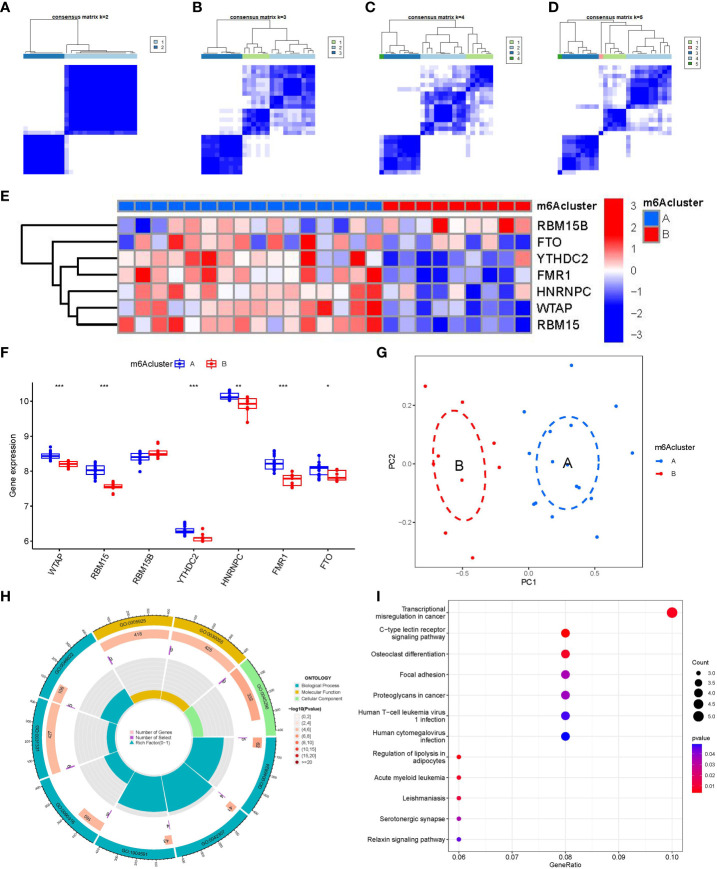
Consensus clustering of the 7 significant m6A modulators in PMOP. **(A–D)** Consensus matrices of the 7 significant m6A modulators for k = 2–5. **(E)** Expression heat map of the 7 significant m6A modulators in clusterA and clusterB. **(F)** Differential expression boxplots of the 7 significant m6A modulators in clusterA and clusterB. **(G)** Principal component analysis for the expression profiles of the 7 significant m6Amodulators that shows a remarkable difference in transcriptomes between the two m6A patterns. **(H, I)** GO and KEGG analysis that explores the potential mechanism underlying the effect of the 90 m6A-related DEGs on the occurrence and development of PMOP. *p < 0.05, **p < 0.01, and ***p < 0.001.

We identified two m6A patterns (clusterA and clusterB) based on the 7 significant m6A regulators *via* the R package of “ConsensusClusterPlus” (**Figures 6A–D**). There were 16 cases in clusterA, and 9 cases in clusterB. Then, we plotted the heat map and histogram, which clearly displayed the differential expression levels of the 7 significant m6A modulators between the two clusters. We observed that the expression levels of RBM15, WTAP, FMR1, FTO, YTHDC2, and HNRNPC in clusterA were higher than those in clusterB, while the expression level of RBM15B exhibited no significant differences between the two cluster (**Figures 6E, F**). The PCA results revealed that the two m6A patterns could be distinguished by 7 significant m6A modulators (**Figure 6G**). We screened totally 90 m6A-associated DEGs between the two m6A patterns, and we carried out GO and KEGG enrichment analyses to excavate the role of these DEGs in PMOP (**Figures 6H, I**). The detailed information of GO and KEGG enrichment analysis was shown in **Supplementary Tables 1, 2**. We observed that GO: 0031331 (positive regulation of cellular catabolic process), GO:0030055(cell-substrate junction), GO:0005925(focal adhesion), and GO:0045296(cadherin binding) were the mainly enriched entries. We finally got totally 12 pathways as shown in **Figure 6I**. These signaling pathways like C-type lectin receptor signaling pathway, and Relaxin signaling pathway may exert regulatory functions on the pathological process of PMOP. Notably, KEGG enrichment analysis showed that osteoclast differentiation was one of the mainly enriched pathways. Specially, several key targets were involved in the pathway of osteoclast differentiation (e.g., RELB, SPI1, LILRA6, TGFB1).

**Figure 7:**
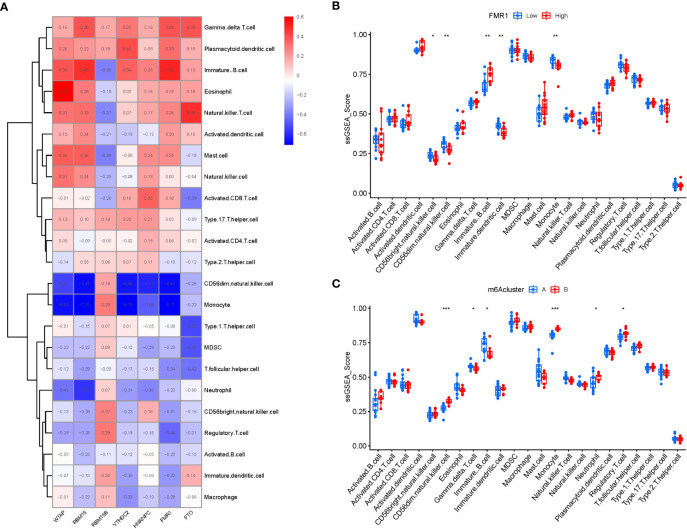
Single sample gene set enrichment analysis. **(A)** Correlation between immune cell infiltration and the 7 significant m6A modulators. **(B)** Difference in the abundance of infiltrating immune cells between high and low FMR1 protein expression groups. **(C)** Differential immune cell infiltration between clusterA and clusterB. *p < 0.05, **p < 0.01, and ***p < 0.001.

Then, ssGSEA was performed to evaluate the immune cell abundance in PMOP samples, and we also assessed the correlation between immune cells and seven important m6A modulators. We observed that FMR1 was positively correlated with many immune cells (**Figure 7A**). We evaluated the differences in immune cell infiltration between patients with high and low FMR1 expressions. The results showed that patients with low FMR1 expression were more likely to exhibit increased immune cell infiltration than those with high FMR1 expression (**Figure 7B**). We found that clusterA was correlated with the immunity of immature B cell and gamma delta T cell while clusterB was related to CD56dim natural killer cell, monocyte, neutrophil and regulatory T cell immunity, indicating that clusterB may be more correlated with PMOP (**Figure 7C**).

### Classification of two distinct m6A gene patterns and construction of the m6A gene signature

**Figure 8:**
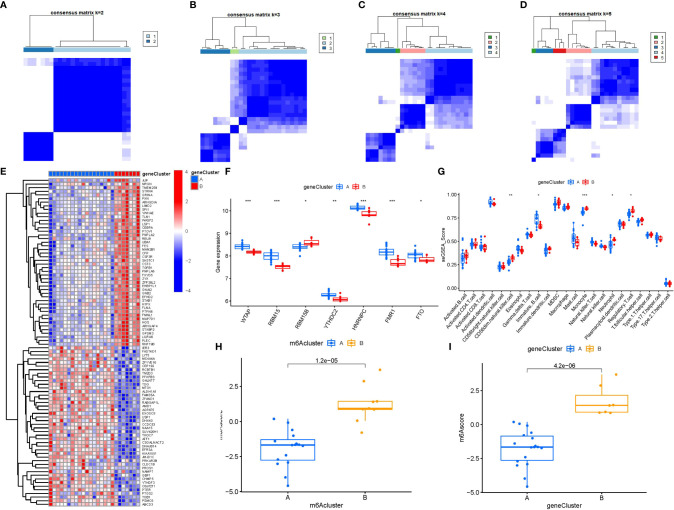
Consensus clustering of the 90 m6A-associated DEGs in PMOP. **(A–D)** Consensus matrices of the 90 m6A-associated DEGs for k = 2–5. **(E)** Expression heat map of the 90 m6A-associated DEGs in gene clusterA and gene clusterB. **(F)** Differential expression boxplots of the 7 significant m6A modulators in gene clusterA and gene clusterB. **(G)** Differential immune cell infiltration between gene clusterA and gene clusterB. **(H)** Differences in m6A score between clusterA and clusterB. **(I)** Differences in m6A score between gene clusterA and gene clusterB. *p < 0.05, **p < 0.01, and ***p < 0.001.

To lucubrate the m6A patterns, we used a consensus clustering approach to classify the PMOP cases into different genomic subtypes on the basis of the 90 m6A-related DEGs. We identified two distinct m6A gene patterns (gene clusterA and gene clusterB), which aligned with the sectionalization of m6A patterns (**Figures 8A–D**). **Figure 8E** displayed the expression levels of the 90 m6A-associated DEGs in gene clusterA and gene clusterB. The differential expression levels of immune cell infiltration and the 7 significant m6A modulators between gene clusterA and gene clusterB were also analogous to those in the m6A patterns (**Figures 8F, G**). These results again verified the veracity of our sectionalization *via* the consensus clustering approach. The m6A scores for each sample between the two distinct m6A patterns or m6A gene patterns were calculated through PCA algorithms for the quantification of the m6A patterns. We found that the clusterB or gene clusterB exhibited higher m6A score than clusterA or gene clusterA (**Figures 8H, I**).

### Role of m6A patterns in distinguishing PMOP

**Figure 9:**
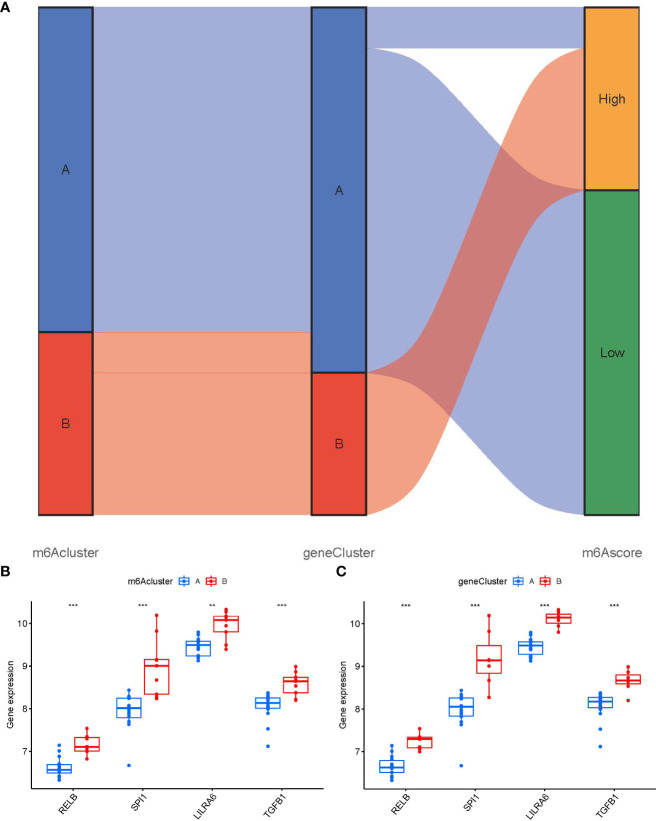
Role of m6A patterns in distinguishing PMOP. **(A)** Sankey diagram showing the relationship between m6A patterns, m6A gene patterns, and m6A scores. **(B)** Differential expression levels of osteoclast differentiation-related genes between clusterA and clusterB. **(C)** Differential expression levels of osteoclast differentiation-related genes between gene clusterA and gene clusterB. **p < 0.01, and ***p < 0.001.

We utilized a Sankey diagram to display the correlation among m6A scores, m6A patterns, and m6A gene patterns (**Figure 9A**). To lucubrate the link between m6A patterns and PMOP, we explored the relationship between m6A patterns and RELB, SPI1, LILRA6, and TGFB1, which were enriched in osteoclast differentiation according to KEGG enrichment analysis. We observed that clusterB or gene clusterB displayed higher expression levels of RELB, SPI1, LILRA6, and TGFB1 than clusterA or gene clusterA, indicating that clusterB or gene clusterB were closely correlated with PMOP characterized by osteoclast differentiation (**Figures 9B, C**).

### RT-qRCR validation of significant m6A modulators

**Figure 10:**
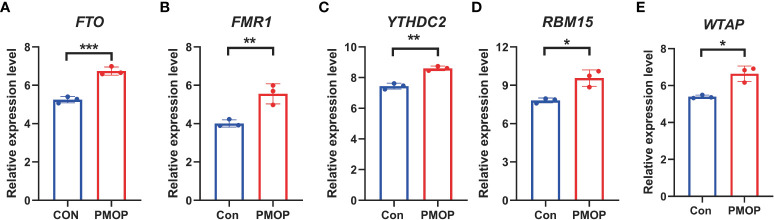
RT-qPCR experimental validation of significant m6A modulators. **(A–E)** Relative mRNA expressions of 5 key m6A modulators including FTO, FMR1, YTHDC2, RBM15 and WTAP between the two groups. All results were expressed as mean ± standard deviation. *p < 0.05, **p < 0.01, and ***p < 0.001.

It was verified that m6A genes FTO, FMR1, YTHDC2, RBM15, WTAP exhibited significantly higher expression levels in PMOP cases than controls (**Figure 10**), which was consistent with the bioinformatics results.

## Discussion

PMOP is a widespread musculoskeletal disorder accompanied by bone system symptoms in postmenopausal women (37). Existing researches have confirmed that m6A modulators play an indispensable role in numerous biological processes (38). However, the role of m6A rmodulators in PMOP stays unclear. This present study aimed at investigating the role of m6A modulators in PMOP.

Firstly, a total of 7 significant m6A modulators were screened from 12 m6A modulators *via* differential expression analysis between controls and PMOP cases, which were selected as diagnostic m6A modulators (FMR1, WTAP, YTHDC2, HNRNPC, FTO, RBM15, and RBM15B) based on an established RF model to predict the occurrence of PMOP. Then, we established a nomogram model on the basis of the seven candidate m6A modulators, which has been evaluated *via* the DCA curve to produce benefit to PMOP patients in virtue of decisions based on the nomogram model.

FMR1 encodes an RNA-binding protein FMRP, which maintains mRNA stability by binding to the m6A site of mRNA (39). Existing study has confirmed that FMR1-deficiency affects skeleton and bone microstructure, demonstrating that knock-out (KO) of FMR1 in mice showed increased femoral cortical thickness, reduced cortical eccentricity, decreased femoral trabecular pore volumes, and a higher range of trabecular thickness distribution compared to controls (40). WTAP (Wilm’s tumor 1 protein) is a ubiquitous nuclear protein that has been reported to facilitate the formation of m6A (41). In addition, existing evidence has confirmed that the WTAP expression level was remarkably upregulated 7 days after fracture (42). Moreover, the increased expression of WTAP has been reported to promote cellular senescence in aging-related diseases (43). YTHDC2 belongs to the DExD/H box RNA helicase family, which exerts important functions in regulating the transcription of mRNA and maintaining the stability of mRNA (44). YTHDC2 knockdown can exert a stimulative effect on the osteogenic differentiation of human BMSCs and suppress the adipogenic differentiation (45). As a DNA binding protein, HNRNPC (Heterogeneous nuclear ribonucleoprotein C) plays an essential part in RNA processing, exerting a remarkably suppressive effect on the transcription of the vitamin D hormone,1,25-dihydroxyvitamin D (1,25(OH)2D) (46). And HNRNPC has properties of species-specific heterodimerization that functions as an indispensable prerequisite for DNA binding and down-regulation of 1,25(OH)2D-related gene transactivation in osteoblasts (47). FTO is a primary m6A demethylase that suppresses osteogenic differentiation by demethylating runx2 mRNA, thus accelerating the process of osteoporosis (48). It has also been found that FTO is a regulator that determines the differentiation of BMSCs by affecting the activation of the GDF11 signaling axis in the bone marrow, promoting Smad2/3 phosphorylation to stimulate osteoclastogenesis and inhibit osteoblast differentiation, thus leading to the development of osteoporosis (49–51). The RNA binding motif protein 15 (RBM15/OTT1) and its paralogue RBM15B (OTT3) belong to SPEN family members (52). Existing studies have confirmed that RBM15 in stress hematopoiesis have a variety of aging-related physiologic changes, including increased DNA damage and NF-κB activation (53), which may serve as important pathological factors in the development of osteoporosis. In addition, study has reported that knockdown of RBM15 and RBM15B impairs XIST-mediated gene silencing (52), which influences osteoblast differentiation in osteoporosis (54). Therefore, to our knowledge, the seven candidate m6A modulators may play an important part in the occurrence and development of osteoporosis according to previous studies.

Existing researches reveal that the dysfunction of T and B ymphocytes may play an essential role in the pathogenesis of PMOP (55). We found that clusterA was correlated with the immunity of immature B cell and gamma delta T cell while clusterB was related to CD56dim natural killer cell, monocyte, neutrophil and regulatory T cell immunity, indicating that clusterB may be more correlated with PMOP (**Figure 7C**). Regulatory T cell (Treg) exerts an essential regulatory function in maintaining immune homeostasis and inhibiting the evolution of PMOP (56). Treg cells negatively regulate osteoclasts in bone metabolism, inhibiting osteoclast formation and differentiation and reducing osteoclast activity (57). The immune and skeletal systems share many regulatory factors, such as transforming growth factor-β (TGFB1), which inhibits osteoclast function of bone resorption and regulates new bone formation in bone resorption region (58). Bozec et al (59) found that Treg cells can regulate osteoclastogenesis by secreting cytokines such as TGFB1, IL-10 and IL-4. In this study, we identified two distinct m6A patterns (clusterA and clusterB) on the basis of the 7 significant m6A modulators as well as two distinct m6A gene patterns (gene clusterA and gene clusterB) based on the 90 m6A-associated DEGs. RELB, SPI1, LILRA6, and TGFB1 were enriched in the pathway of osteoclast differentiation according to KEGG enrichment analysis of the 90 m6A-associated DEGs. ClusterB was closely correlated with the regulatory T cell (Treg) immunity and displayed higher expression levels of RELB, SPI1, LILRA6, and TGFB1, suggesting that clusterB may be linked to osteoclast differentiation. Moreover, the m6A scores for each sample between the two distinct m6A patterns or m6A gene patterns were calculated through PCA algorithms for the quantification of the m6A patterns. We found that the clusterB or gene clusterB exhibited higher m6A score than clusterA or gene clusterA.

Our RT-qPCR experiments verified that m6A genes FTO, FMR1, YTHDC2, RBM15, WTAP exhibited significantly higher expression levels in PMOP cases than controls (**Figure 10**), which was consistent with the bioinformatics results and previous studies. Our results confirm the involvement of these m6A regulators in PMOP and provide new clues to their role in the pathogenesis of PMOP, which further verified the possibility that m6A modulators may play an important role in the development of PMOP. To the best of our knowledge, this study is the first time to report m6A-related diagnostic biomarkers of PMOP in the subtype classification of blood monocytes.

However, there remain some limitations in our study. This study analyzed the relationship between m6A regulators and immune cell infiltration and briefly validated the expression of key m6A regulators in the samples from PMOP patients, but the underlying regulatory mechanisms in the progression of PMOP have not yet been fully elucidated. In the future, more *in vivo*, *in vitro* and clinical experiments are needed to verify the bioinformatics results.

## Conclusion

In general, our present study screened seven diagnostic m6A modulators and constructed a nomogram model providing accurate prediction for the prevalence of PMOP. Then, we authenticatd two m6A patterns based on the 7 m6A modulator, and found that clusterB may be more correlated with PMOP. To our knowledge, this study is the first to report m6A-related diagnostic biomarkers of PMOP in the subtype classification of blood monocytes.

## Data availability statement

The datasets presented in this study can be found in online repositories. The names of the repository/repositories and accession number(s) can be found in the article/**Supplementary Material**.

## Ethics statement

The clinical experiments involved in this paper were authorized by the Ethics Committee of the First Affiliated Hospital of Guangzhou University of Chinese Medicine (No. K[2019]129). The patients/participants provided their written informed consent to participate in this study.

## Author contributions

PZ, HC, DL, JT, JC, ZL, HR, and XJ contributed to the study conception and design. PZ, HC, BX, WZ, and QS contributed to the bioinformatics analysis and experimental validation. JH, GS, XY, ZZ, GZ, GC, and FY contributed to data analysis, and drafting the manuscript. All authors contributed to the article and approved the submitted version.
