# Supplementary material for: Bioinformatics identification and experimental validation of m6A-related diagnostic biomarkers in the subtype classification of blood monocytes from postmenopausal osteoporosis patients
Source: Front Endocrinol (Lausanne). 2023 Mar 8;14:990078. doi: 10.3389/fendo.2023.990078 (PMC10031099; doi:10.3389/fendo.2023.990078)
Supplement: Supplementary file 1 [file DataSheet_1.docx]

Supplementary data

Supplementary Table 1. The detailed information of GO enrichment analysis

| ONTOLOGY | ID | Description | pvalue | p.adjust |
| --- | --- | --- | --- | --- |
| CC | GO:0005925 | focal adhesion | 7.65E-05 | 0.008250454 |
| CC | GO:0030055 | cell-substrate junction | 8.68E-05 | 0.008250454 |
| MF | GO:0045296 | cadherin binding | 1.01E-05 | 0.002581501 |
| BP | GO:0046824 | positive regulation of nucleocytoplasmic transport | 6.71E-06 | 0.013032142 |
| BP | GO:0042307 | positive regulation of protein import into nucleus | 2.77E-05 | 0.021747652 |
| BP | GO:1904591 | positive regulation of protein import | 3.36E-05 | 0.021747652 |
| BP | GO:0090316 | positive regulation of intracellular protein transport | 6.32E-05 | 0.029378761 |
| BP | GO:0031331 | positive regulation of cellular catabolic process | 8.50E-05 | 0.029378761 |
| BP | GO:0046822 | regulation of nucleocytoplasmic transport | 9.08E-05 | 0.029378761 |

Supplementary Table 2. The detailed information of KEGG enrichment analysis

| ID | Description | pvalue | p.adjust |
| --- | --- | --- | --- |
| hsa04625 | C-type lectin receptor signaling pathway | 0.003731602 | 0.236725581 |
| hsa04923 | Regulation of lipolysis in adipocytes | 0.004852055 | 0.236725581 |
| hsa05202 | Transcriptional misregulation in cancer | 0.006433799 | 0.236725581 |
| hsa04380 | Osteoclast differentiation | 0.007772083 | 0.236725581 |
| hsa05221 | Acute myeloid leukemia | 0.007997486 | 0.236725581 |
| hsa05140 | Leishmaniasis | 0.011698755 | 0.288569286 |
| hsa04726 | Serotonergic synapse | 0.033572792 | 0.571839548 |
| hsa04510 | Focal adhesion | 0.034701369 | 0.571839548 |
| hsa05205 | Proteoglycans in cancer | 0.036907494 | 0.571839548 |
| hsa04926 | Relaxin signaling pathway | 0.044782726 | 0.571839548 |
| hsa05166 | Human T-cell leukemia virus 1 infection | 0.047178152 | 0.571839548 |
| hsa05163 | Human cytomegalovirus infection | 0.04914123 | 0.571839548 |
